# Bioinformatics in Italy: BITS2007, the fourth annual meeting of the Italian Society of Bioinformatics

**DOI:** 10.1186/1471-2105-9-S2-S1

**Published:** 2008-03-26

**Authors:** Graziano Pesole, Manuela Helmer-Citterich

**Affiliations:** 1 Dipartimento di Biochimica e Biologia Molecolare - University of Bari, Bari, Italy; 2 Istituto Tecnologie Biomediche del Consiglio Nazionale delle Ricerche, Bari, Italy; 3 Centre for Molecular Bioinformatics, Department of Biology, University of Rome “Tor Vergata”, Rome, Italy

## 

The Italian Society of Bioinformatics (BITS, ) was founded in 2003 from a small group of Italian scientists, involved in disciplines ranging from physics to informatics and molecular biology. The main aims of the Society are the organization of an annual scientific meeting and the fostering of Bioinformatics in Italy. The 4th Annual BITS Meeting, , was held in Naples, Italy, on April 26-28 2007, in the “Federico II” Congress Center, in view of the entire gulf of Naples, the Vesuvio, Castel dell'Ovo, the island of Capri and the city seashore. Over 130 scientists actively working in the field or strongly interested in its development met and discussed their work, state of the art and future perspectives. A total of 149 abstracts were accepted and presented in the poster session and 31 of them were selected for oral presentation.

At the opening of the meeting, Roderic Guigo, Universitat Pompeu Fabra, Barcelona, gave the “Giuliano Preparata” Lecture entitled “Investigation of the signals involved in gene specification in genomic sequences”.

Three keynote talks were given by distinguished scientists: Tom Blundell, University of Cambridge, UK; Christos Ouzounis, Center for Research and Technology Hellas, Greece and Guy Rouleau, University of Montreal, Canada, who gave the “Graziella Persico” Lecture.

The conference was organized into seven thematic sessions: i) Structural and functional analysis of genomes; ii) Novel methodologies, algorithms and tools; iii) Molecular evolution and novel algorithms; iv) Biomedical Informatics; v) Large scale analysis of experimental data; vi) Gene expression and system biology and vii) Structural biology and drug design.

From these conference sessions, 21 papers were submitted for publication in this *BMC Bioinformatics* supplement. We adopted a stringent reviewing procedure, based on selected Associate Editors handling the process according to their recognized knowledge in specific subjects of Bioinformatics and following a peer review procedure.

After this process, 14 papers were accepted. They cover different aspects of theoretical and applied Bioinformatics, including: i) useful algorithms and tools for sequence analysis aimed at the prediction and characterization of functional motifs in nucleotide and protein sequences, of SNPs affecting protein stability, and of protein interaction networks and protein complexes; ii) curated databases of expression data and of conserved non coding sequences; iii) system biology models and iv) interesting technological breakthroughs for data management and for speeding up large-scale database searches.

Whether this will help in encouraging scientists involved in biological and/or medical researches in our country to plan, merge and spread their knowledge by means of Bioinformatics is left to the future and to the availability of national grants specifically devoted to this.

In this presentation, we gratefully acknowledge the patience and help of our colleagues around the world who helped in the process of reviewing as Associate Editors:

Bert Abbot

Gill Bejerano

Axel Brennicke

Idelfonso Cases

Gino Cingolani

Larry Croft

Manolis Dermitzakis

Christiane Gebhard

Jerzy Jurka

Peter Karp

Reinhard Labenbacher

Roman Laskowski

Arthur Lesk

Rodrigo Lopez

John Mattick

Sayan Mukerjee

Annalisa Pastore

Francis Quetier

Peter Rice

Marie-France Sagot

Marco Salvetti

Yves van de Peer

## Next meeting

The next Annual meeting of the Italian Society of Bioinformatics will be held in Cagliari, September 22–26, 2008, in conjunction with ECCB08, the 2008 European Conference of Computational Biology. Further information about BITS2008 will be available on our web site at the address  and at .

